# Medical News and Items

**Published:** 1877-06

**Authors:** 


### Medical News and Items.

### MEDICAL COLLEGE GRADUATES OF 1877.

Jefferson Medical College, 198; University Medical College, N. Y., 157; Ballou Hospital Medical College, 147; University of Pennsylvania, 121; College of Physicians and Surgeons, N. Y., 118; Rush Medical College, 103; University of Louisville, 80; Ohio Medical College, 80; University of Nashville, 70; Louisville Medical College, 47; Albany Medical College, 38; Chicago Medical College, 37; Hospital Medical College, Louisville, 36; Miami Medical College, 35; University of Buffalo, 31; Detroit Medical College, 30; College of Physicians and Surgeons, Indianapolis, 23; Medical College of Indiana, 22; University of California, 20; Medical College of the Pacific, 20; McGill University, Montreal, 19; Woman's Medical College, Philadelphia, 15; Woman's Medical College, Chicago, 4. A grand total of 1,461 doctors turned out in one year!

---

SALE VS. LOUISVILLE MEDICAL COLLEGE.—It will be remembered that last fall L. R. Sale, a student of the Louisville Medical College, (Phenomenon) entered suit against that institution for a return of fees paid in, on the ground that the school was not what it was held out to be. The following, from the Louisville Sunday *Argus*, gives the result of the suit:

The suit of L. R. Sale against the Louisville Medical College, which has been hanging fire in the court of Esquire Speed Peay since last November, was finally decided, by a verdict for the plaintiff, last Thursday. Sale sued for the recovery of fees paid to the concern, claiming that the college was not a legally-constituted institution, inasmuch as there had been great irregularity in the election of the board of trustees. He also made allegations against the efficiency of the school. The court rendered a verdict giving the plaintiff all that he prayed for, thus admitting all the allegations set up by him. This speaks badly for the Louisville Medical College and the Kentucky School of Medicine, (both are the same.) The amount sued for is small, but there is a great question involved in the matter, and that question is, Can the graduates turned out by this school since the election of the present board of trustees (two years ago) be considered diplomated physicians, since the college has been declared not a legally-constituted institution? —*Louisville Med. News.*

---

The Nashville Medical College and the Medical Department of the University of Nashville and Vanderbilt University. These two institutions have two graduating courses every twelve months. They have both broken out this year with the same complaint and are engaged in the much disputed proceeding of graduating two sets of classes every year. A summer session in Nashville must be a very warm affair.

---

The Indiana State Medical Association held a two days session at Indianapolis on the 15th and 16th ult.

The following are the names of the newly elected officers: President, Dr. L. D. Waterman; Vice-President, Dr. N. P. Howard; Secretary, Dr. G. V. Wollen; Assistant Secretary, Dr. G. W. Burton; Treasurer, Dr. J. C. Walker; Librarian, Dr. J. R. Featherstone.

---

The annual meeting of The Chicago Medico-Historical Society was held on the evening of April 30.

The officers for the present year are: President, Dr. W. Godfrey Dyas; Vice-President, Dr. E. L. Holmes; Treasurer, Dr. R. G. Bogue; Secretary, Dr. Plym. S. Hayes; Editor, (permanent) Dr. D. W. Graham. Publication Committee:

Drs. John Bartlett, F. C. Hotz and John E. Owens. Diarist, Dr. S. Wickersham.

At the late meeting of the Illinois State Medical Society a resolution was passed requesting and authorizing the Medico-Historical Society to publish an Annual Medical Register of the State, and directing the co-operation of its own officers in preparing the register.

---

At a late meeting of the Medical Board of Cook County Hospital, Dr. E. Andrews was nominated and recommended for the vacancy on the surgical side of the staff caused by the death of Dr. J. W. Freer. The Board of County Commissioners, however, ignored the nomination, and taking the matter in their own hands appointed Dr. Charles T. Parkes.

Dr. Moses Gunn has recently commenced his first term ot service in that Hospital, he having been appointed on the staff several months ago, in a place created by the remodeling of the rules and regulations of the Medical Board.

---

The Chicago Society of Physicians.—The annual meeting of this society was held Monday evening, May 14. The election for officers resulted as follows: President, Dr. W. H. Byford; Vice-President, Dr. J. H. Etheridge; Secretary and Treasurer, Dr. E. W. Sawyer.

The meetings of the society are held the second and fourth Monday evenings of each month at the Grand Pacific hotel.

---

Mortality in the City of Chicago for the Month ending May 19th, 1877.—Total mortality, 581; males, 311; females, 270; under 5 years of age, 286. The principal causes of death were: Apoplexy, 12; croup, 19; scarlatina, 63; meningitis, 29; convulsions, 52; diphtheria, 12; diseases of heart, 13; phthisis pulmonalis, 74; pneumonia, 38. Population, census

1874, 395,400; estimated population, 420,000; number of deaths to every 1,000, total estimated population, 1.38.

O. C. DeWolf, M. D.,
Commissioner of Health.

---

The members of the medical society of Madison county, Iowa, not wishing to bid against each other for the pauper practice of the county, requested the board of supervisors to make the society, as a body, a proposition for doing the work. The proposition was made and accepted, and the society have undertaken to attend the county patients. The salary received is to be entirely devoted to the establishment of a medical library and reading room. The society hopes to retain the contract for several years, and we hope they may be successful. This is an example which might well be followed by other societies.

---

The Central Free Dispensary of this city has just received a donation of drugs from Henry Thayer & Co., of Cambridgeport, Mass. We are glad to see this worthy institution aided in its efforts to relieve suffering, for although many of our own merchants have ever been generous to this and similar institutions, the officials, who should at least provide medicines for the sick and destitute, are extremely niggardly with these institutions and do not furnish one quarter of the medicines needed by the thousands who apply for medical aid.

---

*Texas Laws and Medical College Diplomas.* The Texas legislature enacted a law last winter, "Regulating the practice of medicine in the State of Texas," providing that all medical men shall be examined by a Board of Examiners, "regardless of their credentials." A letter before us from a Northerner in Texas, a graduate of Rush Medical College, alleges that this law was inspired, and is chaperoned, by medical men of Texas to get, and keep, control of the medical practice of that State.

He appeared before the Board, like a law-abiding citizen, paid the $15 fee exacted, passed his examination, submitted his diploma for inspection, all of which resulted in his receiving through the postoffice "the following characteristic lines: 'We decline to issue to you a certificate of qualification.'" The Doctor claims that the law is a persecution of Northerners, and condemns the high-handed proceeding "as an unwarranted assumption of authority in passing judgment upon the professional qualifications of graduates from properly chartered medical colleges."

---

The Kentucky State Medical Society and the Two Schools of Prof. Gaillard. At its recent annual meeting in Louisville, the largest meeting ever held by this society, the following resolutions were unanimously passed, "with bursts of applause." It would seem that their own State society is up in arms against the one Faculty that conducts two distinct and separate graduating lecture sessions annually, under the extremely gauzy pretext of running two distinct and separate colleges. If his own State society pass resolutions so condemnatory of Professor Gaillard's multifarious annual graduating sessions, how can he expect to make the whole country abstain from questioning the propriety of his course? The resolutions were offered by Dr. John Baker, an ex-president of the society, possessing no college connection whatever. They are as follows:

*Resolved,* That this society is in full accord with the American Medical College Convention, seeking to elevate the Standard of medical education in this country.

*Resolved,* That summer schools, which enable students to graduate after from eight to nine months' study, are exerting an evil influence upon the profession.

*Resolved,* That a winter and summer course by the same school, and graduation at the end of each, tends to deteriorate the standing of the medical profession.

---

The Medical College of Fort Wayne, Ind., issued last year its first annual announcement, containing a Faculty list of ten professorates and two adjunct professorates. Its *raison d'etre* is extremely unique. The diseases peculiar to the Maumee and Wabash valleys furnish the grounds upon which this new college takes its stand. Students should be educated and trained in the part of the country in which they intend to practice. Hence to treat "diseases peculiar to the Maumee and Wabash valleys," a young man must be educated in a college located in those valleys. The colleges of this country ought to take the hint and establish "Professorships of diseases peculiar to the Maumee and Wabash valleys," of the diseases of the far West, of the diseases of the Mississippi basin, of the disease of the upper lakes, and so on *ad infinitum*. The construction of the sentences of this new announcement is something amazing. For example, "There exists in this region forms of diseases peculiar to the country," etc., "It is no longer now as it formerly was" (! !) The following stunning sentences are reproduced *verbatim*, *literatim et punctuatim:*

"To sum up concisely the reasons that have induced so important and arduous an undertaking as the establishment of a new Medical College, we may state that there exists in the region of which Fort Wayne is the geographical center, a well marked endemic influence inducing diseases more or less peculiar to the country.

"That diseases common to other parts also receive a well marked impress from the same endemic influence; that this endemic influence itself undergoes a change, varying from time to time with the history of the country; that the course of the successive stages of endemic influence is dissimilar in different districts, and different in point of time.

"That these facts lead to the inevitable conclusion that every district is to be studied separately, and it becomes every one contemplating the practice of medicine to obtain his clinical instruction in the same district in which he designs to labor."

---

*The Colleges and the Three-Years-Reading Requirement for Graduation.* Every one of the 57 American Medical Colleges, with the exception of the following named schools, require, according to their annual announcements, the three-years-of-study qualification in candidates for the doctorate, viz., The University of Georgia Medical School, The Kentucky School of Medicine, The Louisville Medical College, Central University Medical School, (Louisville) Washington University Medical School, (Baltimore) University of Maryland Medical School, University of Virginia Medical School, Medical College of Virginia and the Medical Department of the University of Nashville and Vanderbilt University. Professor Gaillard is the inspiring genius of the two schools that he is connected with, viz.: The Kentucky School of Medicine and the Louisville Medical College, and he has ceaselessly advocated, for years, the throwing overboard the three-years-study clause, which every physician supposes of vital importance to graduation. He has advocated its abolition, argued for it, fought over it, written upon it, and in every way given himself up to insisting that excellency in examinations was *the* thing to be considered in granting diplomas; and if a young man could stand his examinations at the end of nine months, or of nine years of reading, time made no difference. Every college that he heard of joining him in abolishing the three-years-study clause, he publicly welcomed in his medical journals. No editor in the country has abandoned himself to this new departure in requirements of candidates for graduation so conspicuously as he, and it must be confessed he has wielded a power in the South, when we see that the colleges above enumerated are all Southern schools. The stand that this erudite professor and editor has so conspicuously maintained before the American medical public, is now rendered all the more ridiculous and farcical by a ghastly attempt to show how virtuous his two colleges are, and how recently they have become so. In commenting, in his *American Medical Bi-Weekly*, on the passage of the resolutions adopted by the Kentucky State Medical Society at its late sitting, he submits the following: " The Medical Institution of the State against which these State society resolutions are aimed has had for four months past—(mark well the ancientness of the resolution—FOUR MONTHS!!")—upon its record books a resolution, that no student shall be allowed to come up for graduation who does not file written evidences that he has been a student of medicine for two years; that school (the Kentucky School of Medicine, and one other (the Louisville Medical College) are the only medical institutions in this State which have enacted such a law." For the simple reason, we suppose, that every other medical college in Kentucky requires THREE years instead of two.

The most inconoclastic remarks on graduation qualifications comes from the last announcement of the Medical Department of the University of Virginia. Listen:

"The degree of Doctor of Medicine is conferred upon such students as prove their fitness for the same by rigid and searching examination. It has ever been the policy of the institution to make its honors testimonials of merit, and not certificates of attendance on a prescribed course of instruction. In accordance with this policy, the degree of Doctor of Medicine may be conferred upon a first-course student if found worthy of it. Not only is it within reach of the intelligent, diligent and persevering, to graduate in one session of nine months, but in point of fact many do thus graduate. A longer time, however, is often devoted to the necessary preparation and wisely, when circumstances permit. It is not an unusual case that an academic student, looking forward to medicine as his profession, conjoins a part of the medical with his academic studies during one session; and during the next, entering as a medical student proper, he is enabled to graduate at the close thereof with comparative ease."

The next new wrinkle will be to grant a diploma to students just before they have time to attend lectures.

---

DEATHS OF DOCTORS FROM DIPHTHERIA IN PARIS. M. Carrère has just died of diphtheria, at the early age of thirty-one. He makes the fifth doctor who has died in Paris within a short time from the same disease; viz., MM. Reginauld, Dubois, Mécanden, Cintrat and Carrère. How many others may there be who die obscure and unknown?—*Gar. des Hôpit. Ap.* 24.

